# Altered language network lateralization in euthymic bipolar patients: a pilot study

**DOI:** 10.1038/s41398-022-02202-7

**Published:** 2022-10-06

**Authors:** Zaira Romeo, Marco Marino, Alessandro Angrilli, Ilaria Semenzato, Angela Favaro, Gianna Magnolfi, Giordano Bruno Padovan, Dante Mantini, Chiara Spironelli

**Affiliations:** 1 Department of General Psychology, University of Padova, Padova, Italy; 2 Movement Control & Neuroplasticity Research Group, KU Leuven, Leuven, Belgium; 3 IRCCS San Camillo Hospital, Venice, Italy; 4 Padova Neuroscience Center, University of Padova, Padova, Italy; 5 Department of Neuroscience, University of Padova, Padova, Italy; 6 Unit of Penitentiary Medicine, ULSS6 Padova, Italy

**Keywords:** Neuroscience, Bipolar disorder

## Abstract

Bipolar patients (BD) in the euthymic phase show almost no symptoms, nevertheless possibility of relapse is still present. We expected to find a psychobiological trace of their vulnerability by analyzing a specific network—the Language Network (LN)—connecting many high-level processes and brain regions measured at rest. According to Crow’s hypothesis on the key role of language in the origin of psychoses, we expected an altered asymmetry of the LN in euthymic BDs. Eighteen euthymic BD patients (10 females; age = 54.50 ± 11.38 years) and 16 healthy controls (HC) (8 females; age = 51.16 ± 11.44 years) underwent a functional magnetic resonance imaging scan at rest. The LN was extracted through independent component analysis. Then, LN time series was used to compute the fractional amplitude of the low-frequency fluctuation (fALFF) index, which was then correlated with clinical scales. Compared with HC, euthymic patients showed an altered LN with greater activation of Broca’s area right homologous and anterior insula together with reduced activation of left middle temporal gyrus. The normalized fALFF analysis on BD patients’ LN time series revealed that the Slow-5 fALFF band was positively correlated with residual mania symptoms but negatively associated with depression scores. In line with Crow’s hypothesis postulating an altered language hemispheric asymmetry in psychoses, we revealed, in euthymic BD patients, a right shift involving both the temporal and frontal linguistic hubs. The fALFF applied to LN allowed us to highlight a number of significant correlations of this measure with residual mania and depression psychiatric symptoms.

## Introduction

Bipolar disorder (BD) is a severe, chronic, and heterogeneous psychiatric illness [1, 2] characterized by acute dysfunctional mood states, cyclically switching from depression to mania (BD-I) or hypomania (BD-II) [2, 3], and periods of euthymia—an absence of clinically significant symptoms [4]. While mania comprises elevated or irritable mood, and/or increased goal-directed activity or energy [5], the euthymic phases are associated with mood instability, increased emotional reactivity, and cognitive processing deficits [6].

Despite sharing several characteristics with major depressive disorder (MDD) [7], BD obtained a distinct classification from mood disorders in the DSM-V [8]. Some clinical traits, such as rumination [7], depressive symptoms [7, 9–14], and periods of remission are commonly manifested by both BD and MDD patients. In particular, as BD patients spend more time in the depressive phase than in the manic or hypomanic phase, this could lead to the wrong diagnosis, especially in case of unipolar depression [11]. At the same time, BD also shares genetic and neurobiological traits with schizophrenia (SCZ) [15], as well as psychotic symptoms, including hallucinations and/or delusions, which further increases the chances of misdiagnosis.

When considering together all these characteristics, it becomes clear that BD represents a special condition in which a large variety of symptoms might co-exist depending on the specific phase affecting the patient. This supports the hypothesis on psychosis continuum in which the three described disorders share some neural dysfunctions and the difference would depend on the severity of the disease: thus, SCZ would be at one extreme, MDD on the other and BD in an intermediate position [15, 16]. The psychosis continuum hypothesis has led many authors to propose dimensional approaches instead of a purely categorical distribution of symptoms and pathologies, as, instead, has been proposed in the DSM. Furthermore, the psychotic spectrum continuum hypothesis lays the foundations for a common etiopathogenetic mechanism underlying these disorders. This assumption is the starting point of Crow’s evolutionary theory that suggest a potential common origin of psychoses due to the disruption of left hemisphere dominance for language, typically observed in healthy individuals [17, 18]. Notably, several studies by our group confirmed the presence of language asymmetry alterations using electrophysiological measures in different psychiatric populations [16, 19–21].

In the last years, neuroimaging techniques have been widely used to investigate large-scale networks organization during rest condition in neurological and psychiatric populations [22–25]. In particular, the network approach has proved to be particularly well-suited for investigating the biological basis of psychopathology [26–28]. There is emerging consensus that neuropsychiatric symptoms (i.e., hallucinations, delusions, and depression) could arise from the dysfunction of spatially distributed, interconnected regions [26, 29]. These coherent patterns detected during rest condition, commonly called resting-state networks (RSNs) [30] have been largely studied using functional magnetic resonance imaging (fMRI). With the respect of BD, fMRI data showed altered connectivity patterns of fronto-limbic circuitry, which are linked to different illness phases and mood states [31]. However, recent studies showed heterogeneous results that make the neural correlates of BD still unclear [2]. Aberrant activity has been reported in several brain regions, including anterior cingulate cortex, amygdala and insula, but also in correspondence of areas commonly associated with Default Mode Network, Salience Network, Central Executive Network and Fronto-parietal Network [10, 11, 32–36]. While most of these studies provide a picture on networks’ features in BD during acute phase, very few were dedicated to the investigation of patients’ euthymic phase, during which an apparent remission of symptoms occurs, whose identification of neural correlates to clinical symptoms is even more challenging.

Following Crow’s hypothesis on the common origin of psychoses [17, 18], in the present pilot study we investigated resting-state fMRI features within the Language Network (LN) in a group of BD patients in euthymic phase, to establish whether the presence of alterations in spontaneous network fluctuations might characterize neuropsychiatric diseases [22, 26, 28]. In particular, compared with healthy controls (HCs), we expected to find an altered LN in BD patients, that is, a marked atypical right frontal activation, mainly due to the loss of inhibition from the left language-related dominant Broca’s homologous. This hemispheric alteration would origin from an impaired connectivity of the LN. In this study, we implemented a multi-faceted approach to investigate intrinsic LN features and differences between BD patients in euthymic phase and HCs. This was achieved by using Independent Component Analysis (ICA) to extract LN spatial pattern, and fractional amplitude of low-frequency-fluctuations (fALFF) to assess the spectral content of neuronal activity within LN regions [37, 38] and to potentially identify altered functioning. Finally, we analyzed the association between fALFF alterations within LN regions and the extent of bipolar symptoms assessed with standardized rating scales.

## Material and methods

### Participants

This study has been approved by the Ethics Committee of Padova University Hospital and adheres to the principles of the Declaration of Helsinki. All participants signed the informed written consent before they started their participation in the experiment.

Patients were recruited at the Mood Disorders Outpatient Unit of the Padova University Hospital according to the following inclusion criteria: they received a diagnosis of BD (type I or II) for at least one year, they were non-remitting outpatients, and they were in a euthymic state at the moment of the experimental data collection (Young Mania Rating Scale [YMRS] scores being lower than 8) [39]. Therefore, 18 euthymic BD patients (10 females, average age = 54.50 years, Standard Deviation [SD] = ± 11.38 years) took part in the experiment. Anamnestic and clinical data of BD patients are summarized in Table 1.

**Table 1:** Demographic, anamnestic, and clinical data.

	HC (*n* = 16)	BD (*n* = 18)	Statistics
Age (years)	51.16 ± 11.44	54.50 ± 11.38	*t*_32_ = −0.84
Gender	8 M and 8 F	8 M and 10 F	χ^2^_1_ = 0.10
Height (m)	1.69 ± 0.08	1.68 ± 0.09	*t*_32_ = 0.16
Weight (kg)	69.40 ± 11.21	71.72 ± 8.41	*t*_32_ = −0.69
		**Mean** **±** **SD**	**Range (min–max)**
BD type		I (*n* = 10) and II (*n* = 8)	
Age at onset (years)		32.12 ± 15.79	18–65
Disease duration (years)	21.82 ± 11.08	6–49	
Episodes (*n*)			
Manic		0.82 ± 0.88	0–2
Hypomanic		1.47 ± 1.59	0–6
Depressive		2.82 ± 2.13	1–9
STAI-Y1		38.22 ± 10.25	20–57
STAI-Y2		46.94 ± 8.62	31–59
ASRM		5.33 ± 4.45	0–13
HAM-D		5.50 ± 4.00	0–13
PANAS-PA		29.05 ± 7.75	11–44
PANAS-NA		19.39 ± 7.15	11–31

We also enrolled 16 healthy, age-matched, control participants according to the following exclusion criteria: genetic kinship with some members of the patient group, major lifetime psychiatric diagnosis, and use of psychotropic drugs.

In addition, all of the participants were suitable for MRI scans (e.g., they had no metal bodies in the skull), and none of them suffered from epilepsy and other major neurologic brain comorbidities.

### Clinical assessment

Before the MRI session, participants completed a psychiatric interview (Structured Clinical Interview for DSM-IV) with a board-certified psychiatrist to determine the presence or absence of current and past psychiatric illnesses. At the MRI visit, a psychiatrist completed the YMRS as an eligibility criterion, as well as the Hamilton Depression Rating Scale (HAM-D) [40], Altman Self-Rating Mania Scale (ASRM) [41], and STAI-Y1 and Y2 [42] with BD participants. Higher scores indicate a greater amount of what was measured (e.g., depression, mania, or anxiety). During the same visit, a clinical psychologist administered the Positive And Negative Affective State (PANAS) questionnaire [43]. Patients’ pharmacological treatment has been recorded as well as other features, such as the history of psychotic symptoms, age of onset of BD, duration, mood temporal pattern, and a number of manic, hypomanic, or depressive episodes (details on Table 1).

### MRI data acquisition

Magnetic resonance imaging was carried out at the Radiology Department of Padua University Hospital with a Siemens MAGNETOM® 1.5 T MRI system (Siemens Healthcare, Erlangen, Germany); a specific head coil was mounted to increase the image quality of brain tissues. Participants had both a resting-state fMRI scan during which they were instructed to stay relaxed with their eyes open while remaining motionless (201 continuous functional volumes, repetition time = 2390 ms, echo time = 50 ms, flip angle = 90°, the field of matrix = 64 × 64 × 36, acquisition voxel size = 1.8 × 1.8 × 6 mm^3^; acquisition time 8:00 min) and a high-resolution 3D T1-weighted structural MRI (sMRI) in a gradient-echo sequence (160 sagittal slices, repetition time = 2000 ms, echo time = 3.13 ms, flip angle = 20°, the field of matrix = 320 × 320 × 160, acquisition voxel size = 0.656 × 0.656 × 1 mm^3^; acquisition time 5:33 min). During the functional scan, subjects were asked to simply stay motionless, awake, and relaxed with their eyes open, and not to think about anything in particular. No visual or auditory stimuli were presented at any time during functional scanning. None of the participants in the study moved, fallen asleep, or reported anxiety or other particular emotion during scanning. Preliminarily, all scans were visually inspected by a trained neuroradiologist to exclude gross pathology alterations, excessive motion, or major scanner artifacts.

### MR data preprocessing

fMRI data were preprocessed by means of an automated pipeline developed using SPM12, including motion correction, spatial alignment to sMRI, bias field correction, co-registration to standard space, and spatial smoothing at 6 mm full width half maximum [44, 45].

### LN reconstruction using ICA

Connectivity analysis was performed, separately for each subject, using spatial ICA, which was used for decomposing the fMRI data into brain activity patterns starting from the spatial covariance of the measured signals (as ICA extracts specific patterns from independent sources, this approach takes into account of any physiological confounds, e.g., cardiac and respiratory contributions) [46]. We estimated the number of ICs by using the minimum description length criterion [47]. Accordingly, 45–98 ICs were extracted, depending on the specific fMRI dataset. ICs were calculated using the FastICA algorithm, with a deflation approach and hyperbolic tangent non-linearity [48]. For each IC, a spatial map and an associated time series were extracted. The spatial map shows the intensity of the activity across the voxels of that pattern, whereas the time series corresponds to its course over time [49, 50]. The spatial map was converted to z-scores subtracting the average intensity across voxels, and dividing the resulting map by the standard deviation across voxels. To select a possible independent component of interest (i.e., an independent component associated with a given RSN, such as the LN) for each subject and scan, we used RSN spatial templates from a previous study [44]. The IC corresponding to the LN was identified using an automated template-matching procedure, in which the considered LN template was derived from our previous fMRI study [44]. Specifically, the LN was identified as the IC showing the highest spatial correlation with the LN template map in Montreal Neurological Institute (MNI) space.

### Testing for between-group differences in LN spatial map

Starting from the individual LN spatial map, we derived LN group-level correlation map by performing a one-sample *t* test, using a mass-univariate analysis. According to this approach, each voxel displayed as significant in the results indicates that there was a significant correlation at the group level. We corrected the significance level for multiple comparisons (for multiple voxels involved in the analysis) between single-subject z-scores correlation maps using the Benjamini–Hochberg false discovery rate (BH-FDR) procedure [51], which does not make any assumptions about sample dependency. The significance threshold for the LN group-level correlation map derived from the fMRI data was set to *p* < 0.05, BH-FDR corrected. This was performed separately for each group to visualize the average LN functional connectivity pattern both for the HC and the BD groups. We then performed the LN between-subject comparison between the HC and the BD groups by using a two-sample *t* test (*p* < 0.05, BH-FDR corrected) on the individual LN maps belonging to each group to detect regional differences in the LN map between the two groups.

### Fractional amplitude of low-frequency-fluctuations

Starting from the individual time series associated with the LN, to estimate the fALFF, the frequency spectrum was computed using the Fast Fourier Transform (FFT) function. The fALFF was computed for the whole detectable frequency range, which was subdivided into four separate bands: slow-5 (0.01–0.027 Hz), slow-4 (0.027–0.073 Hz), slow-3 (0.073–0.198 Hz) and slow-2 (0.198–0.25 Hz). This separation was first suggested by Zuo and colleagues [52] to provide better discrimination compared to the canonical fALFF, which is computed in the 0.01–0.1 Hz frequency range. To limit the effect of individual confound, the fALFF values in the four frequency bands were normalized with respect to the canonical fALFF [52, 53].

### Correlation of fALFF with psychiatric and psychological scales

To identify a significant relationship between functional brain measurements and behavioral scores of euthymic BD patients, we performed a correlation analysis between the normalized fALFF frequency band values and the psychiatric and psychological scales administered during the interview.

## Results

### Socio-demographical and clinical data

No significant socio-demographical differences between HC and BD groups were found (all *t* < 1.0). Concerning the BD group, all patients were pharmacologically treated with mood stabilizers, atypical antipsychotics, antidepressants, and anxiolytics (further details in [54]).

### ICA LN spatial maps

**Fig. 1:**
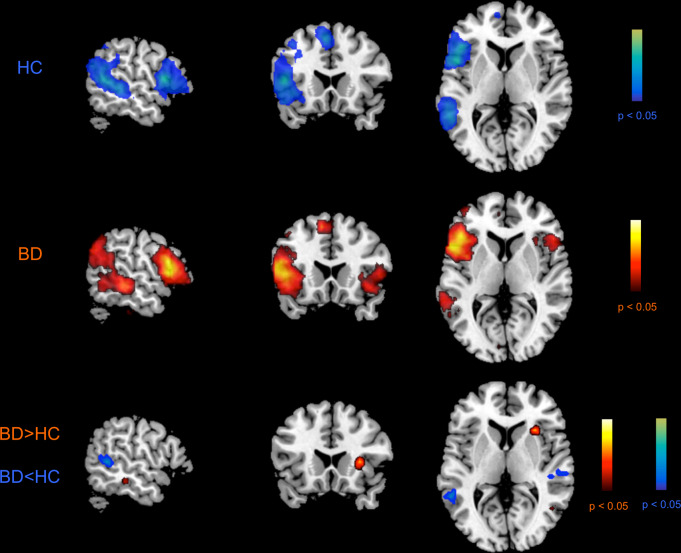
LN maps in HC and BD patients and contrast between groups. Random-effects group-level *t-*maps of the LN in HC (top row, winter color scale) and BD patients (middle row, hot color scale), and the random-effects group-level t-map (all *p*s< 0.05, BH-FDR corrected) for the difference between HC and BD patients (bottom row, hot/winter color scales depending on the group contrast), which was masked to only show the significant differences between the two groups for the LN areas and their homologous. Other regions, apart from the LN, which showed between-group differences are reported in Supplementary Material (Figure S1).

Figure 1 depicts the random-effects group-level *t-*maps for LN of HC (top row) and BD patients (middle row), as well as the random-effects group-level *t*-map for the difference between HC and BD patients (bottom row). Consistent with previous studies on RSNs [50, 55], the LN included activation of most regions in the left hemisphere, that are Broca’s area/frontal operculum (BA 44-45), insula (BA 13), premotor and supplementary motor areas (BA6), angular gyrus (BA 39), superior and middle temporal gyrus (BA 21-22). In addition, in the BD group only, the LN included an atypical extra region located in the right hemisphere (Fig. 1, second row, hot color scale) on the homologous of Broca’s area/frontal operculum (BA 44) and anterior insula (BA 13).

The between-group analyses revealed spatial differences in correspondence to two regions belonging to the LN: compared with HC, BD had significantly greater activation in right insula (BA 13; MNI coordinates: 31, 21,5) but lower activation in left middle temporal gyrus (BA 21; MNI coordinates: −58, −48, 7) and in right primary auditory area (BA 41; MNI coordinates: 57, −27, 8) (Fig. 1, third row, hot and winter color scale, for BD > HC and BD < HC contrasts, respectively).

### LN fALFF analyses

**Fig. 2:**
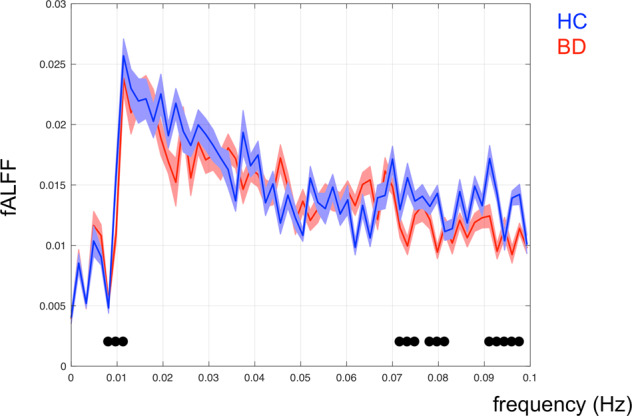
LN fALFF in HC and BD patients. Normalized fALFF analysis carried out on LN time series in BD patients and HC (red and blue lines, respectively).

Considering the normalized fALFF analysis performed on the LN time series, BD patients showed a significantly lower amplitude of their slow fluctuations between 0.007 and 0.0125, 0.07 and 0.0825, and 0.09 and 0.0975 Hz compared to HC (Fig. 2).

**Fig. 3:**
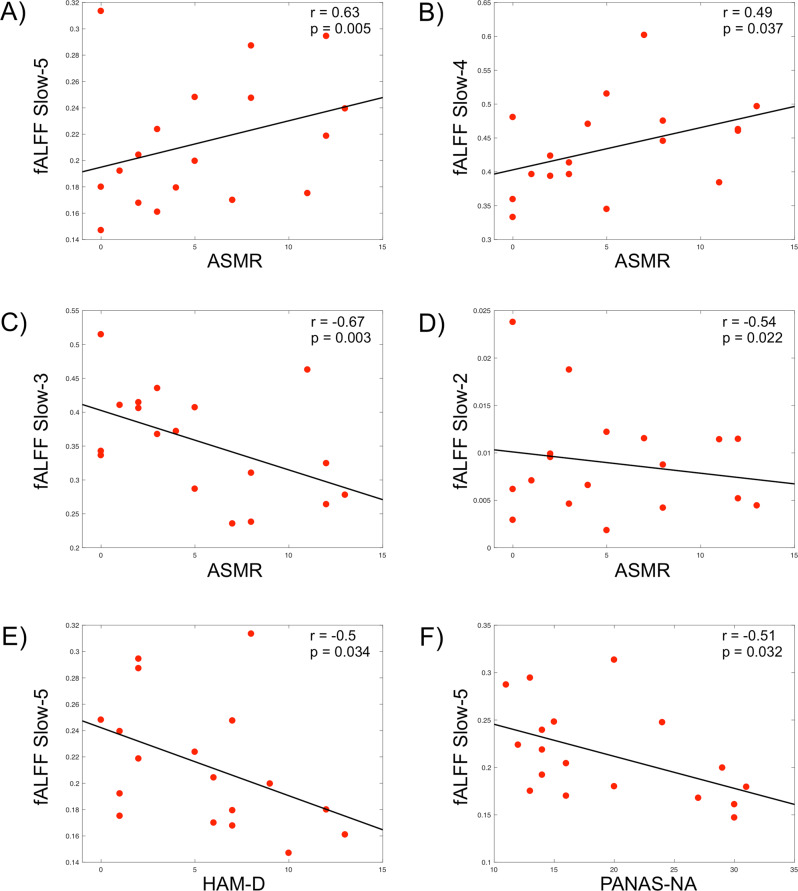
Correlations between LN fALFF in BD patients and clinical symptoms. Significant Pearson correlations between all normalized fALFF bands (LN analysis) and ASMR mania symptoms severity (**A**–**D**), and between Slow-5 and both depressive symptoms severity (HAM-D scores; **E**) and pessimistic attitude (PANAS-NA scores; **F**).

With respect to possible associations with psychiatric/psychological scales, we found that ASMR scores showed significant correlations with all fALFF bands, whereas HAM-D and PANAS-NA scores with Slow-5 only (Fig. 3). Coherently, Slow-5 showed opposite associations with a maniac and depressive symptoms: the higher the spectral content, the greater the severity of mania (Fig. 3A panel), but the lower the depressive symptoms and the negative affect (Fig. 3E, F panels).

The Slow-4 showed the same positive correlation with ASMR manic symptoms as Slow-5 fALFF (Fig. 3B panel), whereas both Slow-3 and Slow-2 revealed a negative association to mania: the lower the spectral content of high-frequency bands, the greater the severity of mania (Fig. 3C, D panels).

### Post hoc regions of interest (ROIs) fALFF analyses

**Fig. 4:**
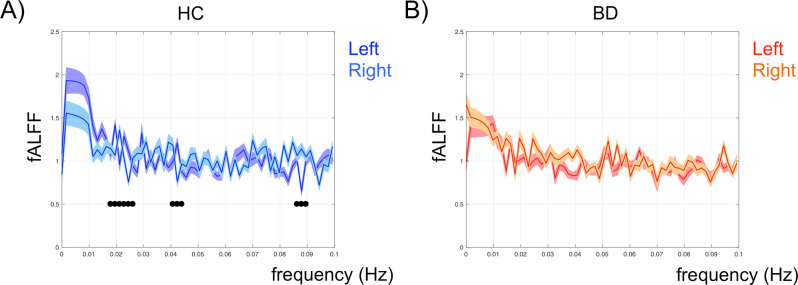
ROI fALFF in HC and BD patients. Normalized fALFF analysis carried out on left and right ROIs time series (MNI coordinates: -54, 21, 6 and 54, 21, 6, respectively) in HC (**A**) and BD patients (**B**) (blue/light-blue and red/orange lines, respectively).

Starting from statistical t maps showing between-group differences, we decided to further carry out an additional analysis focused on BD patients’ greater LN activation in a region that usually is not active thanks to a contralateral inhibition mechanism that ensures left hemisphere dominance for language—the right frontal operculum (homologous of Broca’s area, BA 44). Indeed, according to Crow’s hypothesis on psychosis etiopathogenesis, abnormal linguistic (a)symmetry represents a core risk factor. As BD patients showed significant contralateral activation in the frontal right operculum, including Broca’s area homologous and insula, we set a region of interest (ROI), defined as a 6 mm radius sphere, in this brain region to compare the fALFF spectral power of both this abnormal region (MNI coordinates: 54, 21, 6), as well as the contralateral ROI typically active in LN (MNI coordinates: −54, 21, 6). For each ROI, and separately for each subject, the fALFF was calculated within the defined ROI and then normalized by the mean of the whole-brain fALFF computed across the whole frequency range [56]. More specifically, the ROI-specific fALFF was calculated on a single fMRI signal that was computed, for each ROI, by applying principal component analysis (PCA) on all the fMRI time courses from all voxels included in the spherical ROI [57] and by considering the first principal component as representative of the activity in the whole ROI. Figure 4 shows the fALFF band analysis carried out on these ROIs, in HC (A panel) and BD patients (B panel).

Interestingly, greater left vs. right ROI spectral power was found in HC between 0.0175-0.0275 Hz, and 0.04–0.045 Hz, with greater right vs. left spectral power between 0.085 Hz and 0.09 Hz. Conversely, BD patients showed no asymmetry in ROIs spectral power.

## Discussion

In this pilot study, we tested the continuum hypothesis of a shared neural dysfunction in psychotic spectrum disorders [15, 16] on the one hand and Crow’s hypothesis on the role of language in psychosis on the other. Specifically, we postulated a common origin of psychoses due to ﻿the disruption of left hemisphere dominance for language, as reported for SCZ [17, 18]. Our investigation extends prior work that used a similar methodology by examining euthymic BD patients with minimum residual mood symptoms and no current psychotic symptoms. In past research, this psychiatric population was mainly investigated in relation to RSNs implicated in cognitive control and attention, for which no significant correlations with clinical symptoms were found [2]. In this study, we reported alterations in the LN and we identified the neuronal correlates of LN connectivity with clinical and psychological scales using fALFF measurements. Resting-state fMRI was used to study intrinsic functional alterations involving the LN. We expected to find altered functional connectivity within the LN, which, in line with Crow’s hypothesis, might reflect the underlying pathophysiology of BD as part of the psychotic spectrum continuum. To further investigate whether abnormal functional connectivity patterns are affected by the clinical course of the disease, we also examined the relationship with psychiatric and psychological scales. This was achieved using the fALFF index first at the network level, and then *post hoc* at the ROI level, to focus on a specific region showing an altered spatial pattern in the between-group t maps. Despite the lack of evident symptoms in euthymic patients, we expected to find a potential marker of the hidden disorder by studying an altered LN functioning, that is lower LN fALFF power spectral activity in the left *vs*. right hemisphere. To the best of our knowledge, this is the first study that studied the resting-state properties of LN in BD patients in the euthymic state using fALFF. The combination of information related both to connectivity patterns and the spectral power of neuronal activity might play an important role in the investigation of brain functioning at rest, especially in psychiatric disorders. The integration of the two methods provided a spatial description of LN functioning, together with the abnormal linguistic (a)symmetry, which has been linked to psychosis etiopathogenesis. In line with Crow’s hypothesis [17, 18], we investigated brain functional alterations associated with BD condition in the LN, an issue that received little attention in previous literature. From our results, an altered spatial pattern emerged in the LN. This network encompassed regions in the frontal right operculum, including Broca’s area homologous and insula, which is typically not recruited in healthy volunteers. Concurrently, a significant lack of activation marked BD patients’ posterior left middle temporal gyrus (MTG), a key region within LN associated with several cognitive functions, including language, emotion, memory and social cognition [58–64]. A transcranial magnetic stimulation study revealed that the left posterior MTG plays a critical role in semantic control [63]. Furthermore, MTG alterations were found to be associated with several brain disorders, such as autism spectrum disorder [65, 66], major depression disorders [67, 68], BD [69], and obsessive-compulsive disorder [70]. Notably, a recent study [71] showed decreased connectivity between the left inferior temporal gyrus and the left MTG in SCZ patients with Auditory Verbal Hallucinations (AVHs), compared to those without. These findings suggest that the hypoconnectivity/abnormal connectivity between these areas might be a critical marker of AVHs. Indeed, notwithstanding the considerable heterogeneity of SCZ patients, a number of studies found disrupted connectivity in temporoparietal language areas and primary/secondary auditory cortices in patients with AVHs as compared with SCZ without these symptoms ([72], for systematic review). At the same time, past findings suggested that AVH are associated with increased connectivity among inferior frontal gyrus, Wernicke’s area, and striatal brain regions, also distributed to right hemisphere homologs [72]. Similarly, in the present study we found, in BD patients, an altered LN spatial map not only within left hemisphere regions but also considering an aberrant right hemisphere greater activation, involving right insula/frontal operculum and lower activation of the right primary auditory cortex. Right insula hyperactivation suggests the presence of two frontal linguistic hubs in BD patients making their anterior regions relatively more symmetrical to language and related complex processes—e.g., thought organization and metacognition—that would be impaired during both mania and psychotic active episodes, typically observed in SCZ patients [17–19]. The reduced activity in the right auditory cortex of BD patients, not homologous to the left posterior MTG, revealed a further alteration of the LN. The right temporal regions are involved (together with the left ones) in language comprehension [59], their hypoactivation could be related to the impairment in speech self-monitoring and related BD active maniac symptoms (i.e., tangentiality, derailments, loose associations) and, at neurophysiological level, could arise from an antero-posterior imbalance associated to the increased activation of right operculum/insula inhibiting the posterior sensory areas.

The fALFF analysis was carried out not only on the LN time series of the left hemisphere network, but also in a *post hoc* ROI analysis following the identification of an extra region in the LN spatial map, i.e., the insula/frontal operculum on the right hemisphere, which was not found in the HC group, and confirmed the altered functioning of LN areas in BD patients. In particular, the analysis carried out on the LN time series revealed significantly lower Slow-5 and Slow-3 spectral power in patients than in HCs. In addition, all LN spectral bands have been correlated with mania levels, assessed with ASMR scale, suggesting that the spontaneous fluctuations of this network may represent a physiological marker of patients’ vulnerability to maniac state. Among all bands, the Slow-5 appeared particularly relevant to BD, as the spectral power in correspondence of this band was globally lower with respect to HCs, and showed opposite associations with maniac and depressive symptoms: indeed, the higher the spectral content, the greater the severity of mania, but the lower the depressive symptoms and the pessimistic attitude. According to previous evidence [52, 73], slow-frequency bands (i.e., Slow-5 and Slow-4) represent the activity of gray matter cortical regions, whereas high-frequency bands (i.e., Slow-3 and Slow-2) are mainly originating from deeper regions. In this perspective, it is plausible that Slow-5 spectral power arose from an altered LN, showing opposite associations with BD key symptoms. Concerning the fALFF ROI analysis, left insula lateralization was revealed in Slow-5/Slow-4 bands of controls, but no asymmetry was found in fALFF spectral power of BD patients. Similar to spatial LN maps, also fALFF showed a wider LN activation, spread to both hemispheres, in BD patients. This lack of language asymmetry in prefrontal sites has been found also in SCZ [19] and Major Depression [20] patients, a result in line with the continuum psychosis hypothesis and also with Crow’s theory on the role of an altered language asymmetry on the origin of psychosis [17, 18]. Indeed, all these disorders, in their most severe psychotic expression share several symptoms, including thought disorders, delusions, metalinguistic impairments, all related to altered connectivity among cortical areas and high-level processes [17–21]. As suggested by Crow [17, 18], the impairment of connectivity and hierarchy of the linguistic main hubs, namely hemispheres, and quadrants, increases the vulnerability to consciousness alteration and psychotic disorder onset. As shown here in euthymic bipolar patients, the altered LN asymmetry and hierarchy were also correlated with residual mania and depressive symptoms, for this reason, results are in good agreement with the mentioned models and evolutionistic theory.

Our study has some limitations, especially the limited sample size, which might result in some subtle functional changes in the brain not being statistically detected. Another aspect is represented by the possible confounding effects of medication [74], as almost all of the bipolar patients in our sample were taking medications, including mood stabilizers, antipsychotics, antidepressants, and anxiolytics, which could interfere with the BOLD signal. Nonetheless, we recruited only patients in the euthymic phase and we carried out the resting-state analyses using a data-driven approach that allowed achieving a global view on LN functioning, both in terms of functional connectivity and spectral features. Notably, our approach allowed also correlating neuroimaging measures with clinical scores, showing the suitability of the proposed analysis for investigating the psychopathology of other psychiatric disorders, in line with the need to test the psychotic spectrum continuum hypothesis.

Past studies identified in psychotic BD patients a series of networks that presented aberrant connectivity patterns, which were shared with SCZ patients, suggesting that common psychotic symptoms exist between the two clinical groups [75]. Resting-state analysis can be easily applied to almost all clinical populations since it does not require participation in a voluntary task. Thus, the present methodology could also be applied to investigate SCZ and MDD patients for achieving a comprehensive overview of the differences and shared traits, among all these disorders and test the existence of a continuum in which each phase might be characterized by specific spatial patterns and spectral signatures. Future research is warranted to study specific brain regions and networks for which aberrant connectivity may be reflective of psychosis in general, such as the insula which has been linked to psychotic symptoms both in MDD [76] and in BD [77].

## Supplementary information

Supplementary material

## Data Availability

All the codes used to generate results in this study can be requested from the corresponding author, for academic purposes only.
