# Supplementary material for: Altered language network lateralization in euthymic bipolar patients: a pilot study
Source: Transl Psychiatry. 2022 Oct 6;12:435. doi: 10.1038/s41398-022-02202-7 (PMC9537562; doi:10.1038/s41398-022-02202-7)
Supplement: Supplementary file 1 — Supplementary material [file 41398_2022_2202_MOESM1_ESM.docx]

**SUPPLEMENTARY MATERIAL**

***Altered language network lateralization in euthymic bipolar patients: A pilot study***

*Zaira Romeo, Marco Marino, Alessandro Angrilli, Ilaria Semenzato, Angela Favaro, Gianna Magnolfi, Giordano Bruno Padovan, Dante Mantini and Chiara Spironelli*

**Figure S1** of the Supplementary Material depicts the random-effects group-level t-maps for LN of HC (top row) and BD patients (middle row), as well as the unmasked random-effects group-level t-map for the difference between HC and BD patients (bottom row). The mask used for displaying Figure 1 in the main text of the manuscript was defined by using – as reference – the LN-template which was used for the template-matching procedure used to identify the LN component for each subject. This template was binarized and summed to its corresponding projection into the right side of the brain in order to include the homologous region of the LN. This was done to give a major focus in the main text on the LN. However, as shown in Figure S1 of the Supplementary Material, other regions apart from the LN showed between group differences: compared with HC, BD had significant greater activation in the right visuo-associative area (BA 19; MNI coordinates: 11, -63, 6) and right ventral posterior cingulate cortex (BA 23; MNI coordinates: 27, -62, 6) but lower activation in left orbitofrontal cortex (BA 10; MNI coordinates: -7, 60, 10) (Figure S1, third row, hot and winter color scale, for BD>HC and BD<HC contrasts, respectively).


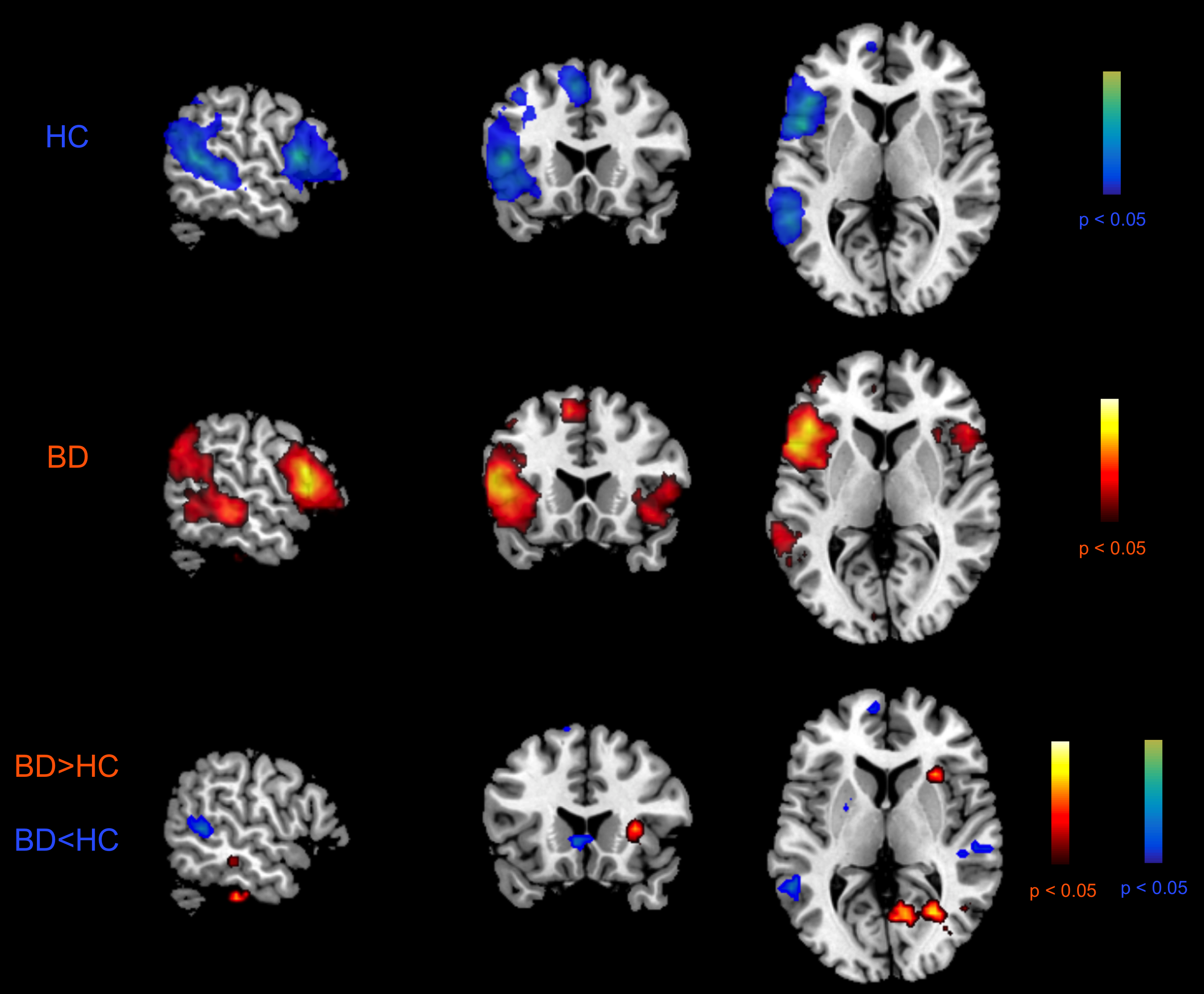


**Figure S1.** Random-effects group-level t-maps of the LN in HC (top row, winter color scale) and BD patients (middle row, hot color scale), and the random-effects group-level t-map for the difference between HC and BD patients (bottom row, hot/winter color scales depending on the group contrast).

Notably, some clusters not belonging to the LN and not present in the threshold spatial pattern of the BD group, emerged from the BD>HC and the BD<HC contrasts. In particular, activity of two regions (the right visuo-associative area and the right ventral posterior cingulate cortex), which are respectively involved in visual information processing ^1^ and in monitoring the focus of attention ^2^, was not found in the HC group, but only emerged in the unthresholded BD group spatial pattern. By contrast, one cluster showed decreased activity in BD group compared to controls. This region corresponds to the left anterior prefrontal/orbitofrontal cortex commonly associated to episodic remembering ^3^, respectively. Still, these clusters did not survive the statistical test at p<0.05 in the random-effects group-level t-map, and their presence will not be further discussed as not belonging to the conventional LN areas.

**REFERENCES**

1. Zeki S. The visual association cortex. *Curr Opin Neurobiol*. 1993;3:155-159. doi:10.1016/0959-4388(93)90203-B

2. Leech R, Sharp DJ. The role of the posterior cingulate cortex in cognition and disease. *Brain*. 2014;137(1):12-32. doi:10.1093/brain/awt162

3. Ranganath C, Johnson MK, D’Esposito M. Left anterior prefrontal activation increases with demands to recall specific perceptual information. *J Neurosci*. 2000;20:1-5. doi:10.1523/jneurosci.20-22-j0005.2000
